# Phenibut (*β*-Phenyl-*γ*-aminobutyric Acid) Dependence and Management of Withdrawal: Emerging Nootropics of Abuse

**DOI:** 10.1155/2018/9864285

**Published:** 2018-04-30

**Authors:** Tania Ahuja, Ofole Mgbako, Caroline Katzman, Allison Grossman

**Affiliations:** ^1^Department of Pharmacy, New York University Langone Health, 550 First Avenue, New York, NY 10016, USA; ^2^Department of Medicine, New York University Langone Health, 550 First Avenue, New York, NY 10016, USA; ^3^New York University School of Medicine, 550 First Avenue, New York, NY 10016, USA; ^4^Department of Psychiatry, New York University Langone Health, 550 First Avenue, New York, NY 10016, USA

## Abstract

This case report describes the development of withdrawal from phenibut, a gamma-aminobutyric acid-receptor type B agonist. Although phenibut is not an FDA-approved medication, it is available through online retailers as a nootropic supplement. There are reports of dependence in patients that misuse phenibut. We report a case in which a patient experienced withdrawal symptoms from phenibut and was successfully treated with a baclofen taper. This case report highlights the development of phenibut use disorder with coingestion of alcohol and potential management for phenibut withdrawal. We believe clinicians must be aware of how phenibut dependence may present and how to manage the withdrawal syndrome.

## 1. Background

Phenibut (*β*-phenyl-*γ*-aminobutyric acid) is a gamma-aminobutyric acid subtype b (GABA-B) agonist that has both anxiolytic and nootropic activity. Phenibut has been available since the 1960s and is available over-the-counter as a supplement marketed for depression, anxiety, and posttraumatic stress disorder in many parts of the world [1]. Furthermore, it is available without a prescription in retail stores mostly in powder form. Although structurally similar to baclofen (*β*-(4-chlorophenyl)-GABA), another GABA-B agonist and a commonly prescribed muscle relaxant, it is 30 times less active [1]. In addition, similar to the mechanism of action of gabapentin, a commonly used medication for neuropathic pain, phenibut may decrease the activity of voltage-dependent calcium ionic channels [1, 2].

Tolerance may develop with long-term phenibut use and dosage increases may result in significant side effects. In addition, abrupt discontinuation of phenibut may result in withdrawal which can be severe and require hospitalization. Physical and psychological withdrawal symptoms include anxiety, agitation, decreased appetite, depression, cognitive deficit, fatigue, dizziness, palpitations, insomnia, nausea/vomiting, and tremors. Psychotic symptoms such as auditory/visual hallucinations, disorganization, and delusions have also been reported [3].

Currently, few studies explore the optimal management of phenibut toxicity and withdrawal. Here, we discuss a case of phenibut withdrawal and options for pharmacologic therapy.

## 2. Case Presentation

A 21-year-old man with a history of anxiety and alcohol abuse presented with three days of insomnia, visual hallucinations, and worsening anxiety after a one-week-long binge of alcohol and concurrent phenibut use. He reported a four-year history of week-long alcohol binges followed by brief periods of sobriety; however, he denied ever experiencing symptoms of alcohol withdrawal. The patient first purchased powdered phenibut from an online retailer several months prior to presentation, reportedly in order to alleviate his anxiety and help him to focus on his schoolwork. He took about one scoop (100–300 mg) as labeled on the product he had, every few days. During an alcohol binge one week prior to admission, the patient began adding one scoop of phenibut to each alcoholic beverage in escalating doses. The night prior to admission, he stated that he used “much more phenibut than usual,” although an exact quantity is unknown. A few hours later, the patient felt increasingly anxious and tremulous and reported diaphoresis, insomnia, and visual hallucinations including dragons, flashes of color, and disturbing sexual imagery. He attempted to vomit to remove the drug from his system and, after an unsuccessful attempt, presented to the emergency department for further evaluation.

On presentation to the emergency department, he appeared distressed and anxious. He was afebrile, oxygen saturation was 100% on ambient air, his blood pressure was 157/83 mmHg, and heart rate was 80 bpm. He was observed in the emergency room and prescribed chlordiazepoxide 25 mg × 4 doses given his recent alcohol binge. He was admitted to the medicine service for further management of his symptoms.

On admission to the medical floor, the patient continued to report visual hallucinations and feelings of overwhelming anxiety. However, when utilizing the Clinical Institute Withdrawal Assessment of Alcohol Scale, Revised (CIWA-Ar) [4], he had no evidence of nausea and vomiting, tremor, paroxysmal sweats, and tactile or auditory disturbances, and he was fully oriented. Given his symptoms and escalating doses of phenibut prior to presentation, phenibut withdrawal was suspected over alcohol withdrawal. He was switched to a baclofen taper with a starting dose of 5 mg three times a day for two days. This dose was deescalated over four days to 5 mg in the morning and 5 mg in the evening, 5 mg in the morning and 2.5 mg in the evening, then 2.5 mg twice a day, then 2.5 mg once a day, and finally cessation of the medication. With baclofen treatment, the patient reported improvement in his anxiety and complete remission of his visual hallucinations and his blood pressure normalized.

The patient tolerated the baclofen taper without side effects, with resolution of his withdrawal symptoms, and was discharged on hospital day 5. He returned to cognitive behavioral therapy seven days after discharge and reportedly did not consume phenibut on follow-up visit three months after hospitalization.

## 3. Discussion

Phenibut withdrawal may mimic benzodiazepine withdrawal with symptoms including anxiety, insomnia, and tension [5]. Baclofen has been an attractive medication for phenibut withdrawal given its similar molecular structure and mechanism of action. At least one case report has suggested treating with 10 mg of baclofen for every 1 gram of phenibut [6]. Chronic phenibut use at high doses may require a slow recovery period up to six months; some case studies have demonstrated prolonged treatment with baclofen for phenibut withdrawal [5, 7]. Yet another case report described the use of phenobarbital as a treatment modality for phenibut withdrawal in a young patient who developed anxiety, tremor, cravings, and muscle aches after stopping use of phenibut [8].

The pharmacokinetics of phenibut are not well elucidated; however phenibut appears to rapidly distribute to the brain, kidneys, liver, and urine with an elimination half-life of 5.3 hours [1, 2]. Of note, due to the addition of a phenyl group, phenibut easily passes through the blood-brain barrier and binds to the GABA-B receptor, leading to psychotropic inhibitory actions [1]. Our team managed the acute withdrawal with a shorter course of medication, as this patient's duration of abuse was shorter than other published cases.

Interestingly, although case reports have mentioned concomitant alcohol abuse, there is limited information available on the pharmacokinetics of phenibut and how best to treat the withdrawal. Further studies are necessary to understand the best pharmacologic approach to phenibut withdrawal and to characterize the specific challenge of coingestion of alcohol and phenibut. Given that our patient had never experienced alcohol withdrawal despite chronic alcohol use and these symptoms emerged with escalating phenibut use, we suspected the symptoms to be related to the phenibut abuse rather than alcohol withdrawal. It remains unclear how alcohol and phenibut each potentiate the effect of the other.

Despite the patient initially receiving chlordiazepoxide for presumed alcohol withdrawal symptoms, we felt that the patient's presentation, short half-life of phenibut, and time course of our patient's substance abuse were more consistent with an acute phenibut withdrawal syndrome. Due to the structural similarity to phenibut, and previously published case reports, we attempted to use baclofen as a taper for our patient. Baclofen, a GABA-B agonist, similar to phenibut, blocks voltage-gated calcium channels; however it does not have affinity for the gamma-hydroxybutyrate (GHB) receptor; therefore abuse potential is minimal [9]. Further, it is more potent by weight as an agonist of the GABA-B receptor. The use of other treatment modalities such as phenobarbital or gabapentin was not utilized for the patient, to avoid toxicities such as sedation, potential therapeutic drug monitoring, and slow onset of these agents. Given the patient's acute binge of alcohol and phenibut, we felt a one-week completed taper was a reasonable duration with close outpatient follow-up.

This case highlights the importance of management approaches for both toxicity and withdrawal of increasingly used substances readily available to the public such as phenibut. Further studies are necessary to understand the best pharmacologic approach to phenibut withdrawal and to characterize the specific challenge of coingestion of alcohol and phenibut.
